# Cross-system transfer of fatty acids from aquatic insects supports terrestrial insectivore condition and reproductive success

**DOI:** 10.1007/s00442-025-05827-9

**Published:** 2025-11-12

**Authors:** Catrin F. Eden, Richard K. Broughton, Bart Donato, Chris M. Hewson, Caroline Isaksson, Stuart P. Sharp

**Affiliations:** 1 Lancaster Environment Centre, Lancaster University, Library Ave, Bailrigg, Lancaster, LA1 4YQ UK; 2 UK Centre for Ecology & Hydrology, Wallingford, OX10 8BB UK; 3 Natural England, Wayfaring House, Murley Moss, Oxenholme Rd, Kendal, LA9 7RL UK; 4 British Trust for Ornithology, The Nunnery, Thetford, Norfolk, IP24 2PU UK; 5 Department of Biology, Lund University, 223 62 Lund, Sweden

**Keywords:** Aerial insectivores, Aquatic subsidies, Eco-physiology, Food quality, Omega-3 highly unsaturated fatty acids

## Abstract

Cross-system fluxes of aquatic insects rich in omega-3 long-chain polyunsaturated fatty acids (ω-3 LC-PUFAs), specifically eicosapentaenoic acid (EPA) and docosahexaenoic acid (DHA), may subsidise insectivorous vertebrates that are suffering due to terrestrial insect declines. The benefits of high ω-3 LC-PUFA diets, such as improved growth and immunocompetence, have previously been demonstrated for riparian insectivores. Understanding the potential for aquatic insects to benefit a range of species is necessary for informing land management practices. Using the spotted flycatcher (*Muscicapa striata*), a habitat-generalist, insectivorous bird as a model, we tested how blood plasma ω-3 LC-PUFA concentrations and the ratio of ω-6:ω-3 fatty acids were related to the body condition of 14 adults and 84 chicks in the wild. We measured how variation in ω-3 LC-PUFAs and ω-6:ω-3 was related to river proximity, to test whether they were related to aquatic insect availability. We assessed how these relationships may extend beyond the individual level by monitoring flying insect availability throughout two breeding seasons and testing its ability to predict reproductive success. EPA was positively correlated with body condition in chicks and adults and declined with distance from a river. Conversely, ω-6:ω-3 was negatively correlated with body condition and increased with distance from a river. Breeding success was positively correlated with aquatic insect availability, suggesting that access to a high ω-3 LC-PUFA diet has the potential to mitigate declines in generalist insectivores. Our results highlight how the maintenance or creation of high-quality freshwater habitats may be promoted in a conservation context for non-riparian species.

**Supplementary Information:**

The online version contains supplementary material available at 10.1007/s00442-025-05827-9.

## Introduction

The flow of nutrients from aquatic to terrestrial habitats may provide an important, yet often overlooked, resource for terrestrial insectivores. Whilst the exchange of insects between aquatic and terrestrial habitats has been well studied (Polis et al. 1997; Baxter et al. 2005; Schindler and Smits 2017), and numerous terrestrial insectivores are known to consume aquatic invertebrates (Baxter et al. 2005; Hagar et al. 2012; Kautza et al. 2016), the importance of these aquatic subsidies at the individual and population levels is poorly understood, particularly for species that are not riparian specialists. For terrestrial, insectivorous birds and mammals, aquatic habitats offer a relatively high abundance of insect biomass and a greater temporal availability of insects due to the addition of emergent aquatic insects and their asynchronous peaks (Nakano and Murakami 2001; Uesugi and Murakami 2007; Kautza et al. 2016; Twining et al. 2018b; Génier et al. 2021). Some bird species that take advantage of these subsidies are explicitly riparian and rely heavily on aquatic subsidies, whilst others move into these areas in response to ephemeral insect emergences (Gray 1993; Elgin et al. 2020). Hence, quantifying the contribution of aquatic systems to the fitness of terrestrial insectivores could improve our understanding of food webs and the extent to which restoration or creation of aquatic habitats should be considered for these species.

In addition to supplementing insect quantities, aquatic subsidies also carry nutritional benefits. Aquatic primary producers, which are consumed by aquatic insects, contain high levels of omega-3 long-chain polyunsaturated fatty acids (ω-3 LC-PUFAs), specifically eicosapentaenoic acid (EPA) and docosahexaenoic acid (DHA; Parmar et al. 2022). This is in contrast to most terrestrial primary producers that are only able to synthesise the ω-3 LC-PUFA precursor alpha-linolenic acid (ALA), which is an essential nutrient, i.e. cannot be synthesised endogenously (Gladyshev et al. 2013). Demand for dietary ω-3 LC-PUFAs is dependent on the endogenous ability to elongate ALA into EPA or DHA, which is consequently determined by evolutionary history and trophic niche. For example, many aquatic animals with reliable access to dietary ω-3 LC-PUFAs have limited capacity to synthesise them from ALA, whereas terrestrial generalists have a greater capacity for conversion (Twining et al. 2016a). Nonetheless, dietary acquisition of ω-3 LC-PUFAs may be favourable in any circumstance due to the energetic cost of conversion (Twining et al. 2018a).

ω-3 LC-PUFAs have a number of important physiological roles, including the maintenance of neuronal membranes, immune function, and anti-inflammatory responses (Larsson et al. 2004; Twining et al. 2016a). DHA has a vital role in brain development and functioning, whereas EPA, the most abundant ω-3 LC-PUFA in aquatic insects (Parmar et al. 2022), is an important precursor to DHA (Dyall 2015). Deprivation of ω-3 LC-PUFAs has been shown to negatively influence a range of physiological functions in humans and other terrestrial and aquatic animals, including brain development and vision (Birch et al. 1998; Chen et al. 2012; Brenna and Carlson 2014; Twining et al. 2016b, 2019). In addition to the absolute concentration of ω-3 LC-PUFAs, the quantity of ω-6 fatty acids relative to ω-3 fatty acids (ω-6:ω-3) has important implications for health. ω-6 fatty acids dominate in terrestrial food webs, to the extent that the ω-6:ω-3 signature can be used to differentiate between aquatic and terrestrial diets (Koussoroplis et al. 2008). The ω-6 fatty acid linoleic acid (LA) has the potential to decrease ω-3 LC-PUFA synthesis by competing with ALA for enzymes and transporting systems (Brenna et al. 2009). For example, domestic chickens (*Gallus gallus domesticus*) fed higher ω-6:ω-3 diets showed lower ω-3 LC-PUFA synthesis and deposition compared with those fed a low ω-6:ω-3 diet (Ibrahim et al. 2018). In addition, the balance of ω-6 and ω-3 fatty acids can determine levels of inflammation and oxidative stress by mediating the balance of pro-inflammatory (ω-6) and anti-inflammatory (ω-3) molecules (Larsson et al. 2004; Isaksson 2015).

Terrestrial insects are reported to be in decline globally (Hallmann et al. 2017; Sánchez-Bayo and Wyckhuys 2019), but whilst some studies report an increase in aquatic invertebrates (van Klink et al. 2020), very few studies have compared the relative abundance of the two groups at the same site. For declining generalist insectivores, more research is needed to establish the potential for the relative quantity or quality of aquatic invertebrates to offset the impact of decreases in terrestrial insects. The capacity for dietary ω-3 LC-PUFAs to benefit insectivorous species is dependent on their ALA to ω-3 LC-PUFA conversion efficiency; those with high efficiency may not be limited by ω-3 LC-PUFAs as they may acquire enough via ALA conversion. For example, tree swallow (*Tachycineta bicolor*) and Eastern phoebe (*Sayornis phoebe*) chicks have been experimentally shown to benefit from increased ω-3 LC-PUFA diets, which resulted in improved growth, immunocompetence and metabolism (Twining et al. 2016b, 2019). However, both of these species are commonly associated with riparian habitats (Schukman et al. 2011; Twining et al. 2019), and so the benefits observed may be due to high reliance on dietary ω-3 LC-PUFAs and consequential low ALA to ω-3 LC-PUFA conversion efficiency. In contrast, a study of blue tits (*Cyanistes caeruleus*), a generalist species found in both aquatic and terrestrial habitats, concluded that nestlings were not limited by dietary ω-3 LC-PUFA availability due to their capacity to synthesise DHA from ALA, though the physiological impact of higher ω-3 LC-PUFA diets on nestlings was not reported (Twining et al. 2021). Hence, understanding if the advantages of dietary ω-3 LC-PUFA acquisition are limited to riparian species is key to determining whether aquatic subsidy provision might benefit a wider range of species.

So far, our understanding of the fitness benefits of ω-3 LC-PUFAs in insectivorous birds has been driven by experimental laboratory studies, and those linking aquatic subsidies with reproductive success are limited to correlational studies. For example, breeding success in tree swallows was positively correlated with the availability of ponds and aquatic insects, but not terrestrial insects (Twining et al. 2018b; Berzins et al. 2021), and aerial songbird abundance in the US was positively correlated with aquatic insect emergence (Schilke et al. 2020). However, more detailed studies are required to determine whether such correlations have arisen from the nutritional subsidies provided by aquatic habitats or by habitat complexity and other confounding factors.

In this study, we investigate the importance of aquatic subsidies in wild birds, specifically for a species that is not frequently associated with freshwater habitats. By doing so, we aim to investigate whether providing high-quality aquatic habitats could be beneficial for terrestrial species. We used the spotted flycatcher (*Muscicapa striata*) as a model species, owing to its strict insectivorous diet but use of both aquatic and terrestrial habitats (Cramp and Perrins 1993; Stevens et al. 2007). We assessed how chick and adult body condition varied with fatty acid composition, specifically the ω-3 LC-PUFAs EPA and DHA, and ω-6:ω-3. To ensure that the fatty acid concentrations observed were a result of diet rather than synthesis, we also investigated how river proximity was related to fatty acid composition. Finally, to understand whether the individual effects were sufficient to influence productivity, we examined how breeding success varied with the availability of aquatic insects. We predicted that higher ω-3 LC-PUFA and lower ω-6:ω-3 would result in better body condition of spotted flycatchers. We expected that ω-3 LC-PUFA concentrations would decrease and ω-6:ω-3 would increase for nests further away from rivers, which would imply that the composition was being driven by aquatic insects. Finally, we predicted that the availability of aquatic insects would be positively correlated with breeding success.

## Methods

### Study species

The reliance of spotted flycatchers on flying insects, alongside rapid insect declines, may be associated with the species’ 93% decline in the UK since the 1960s (BTO 2024) and 56% decline across its European breeding range (PECBMS 2025). Spotted flycatchers frequently occur along riverbanks with suitable perch sites and use riparian and wetland areas on their wintering grounds in Africa (Cramp and Perrins 1993). We have previously shown that spotted flycatcher occupancy in the UK was positively correlated with the density of rivers in 2 km × 2 km squares, but the mechanism behind this relationship was unclear (Eden 2025). However, they are not exclusively riparian and can be found in a range of wooded and semi-wooded habitats, including parkland, farmland and woodland (Stevens et al. 2007). During breeding, most foraging trips occur within 50 m of the nest, but occasional flights of up to 200 m can occur during egg incubation (Davies 1977).

### Nest monitoring and sampling

Fieldwork was undertaken within an 11 km radius of Sedbergh, in the Yorkshire Dales National Park, UK (54.323559, − 2.528300) (Fig. S1). The study area is primarily upland pasture, grazed by livestock, with frequent farmsteads, villages, small pockets of woodland and numerous streams and small rivers. We monitored 128 spotted flycatcher nesting attempts between May and August in 2023 (*n* = 63) and 2024 (*n* = 65) (Fig. S1). GPS coordinates of nests were later used to calculate the distance to the nearest river using ArcGIS and the CEH river networks dataset (UKCEH 2000). We searched for nests in all areas where flycatcher pairs were detected. We recorded the date the first egg was laid (first egg date (FED)), clutch size (full clutches only), hatch date, number of hatchlings, fledging success and the number of chicks present 1–3 days prior to fledging for all nests (where possible). Dates were back-calculated for nests found at later stages, e.g. during incubation or with chicks present, based on an incubation period of 13 days starting from the day the final egg was laid. Nests were checked at least once per week, and, to ensure accurate ageing of nestlings, nests were checked every other day when found during incubation, or when the estimated hatch date was imminent. Chicks were aged ± 1 day based on body size and feather development. At 7 days old (hatch day = day 0), each chick was weighed ± 0.1 g using an electronic balance and tarsus length was recorded ± 0.1 mm by measuring from the inter-tarsal joint to the joint between tarsus and toes using dial callipers. Adults were captured using a combination of mist nets and spring-loaded ‘perch traps’ at varying stages of nesting, and biometrics were measured the same way as for chicks. British Trust for Ornithology metal rings were fitted to all chicks and adults, and a unique combination of two or three anodised-metal colour rings was fitted to each adult. Biometric measurements were later used to calculate a scaled body mass index (SMI; body condition from hereon in) by standardising mass to the mean mass of sampled birds with a scaling exponent estimated from standardised major axis (SMA) regression of mass against tarsus length (Peig and Green 2009). In 2023 only, up to 100 μL of blood was extracted from the brachial vein of adults and chicks with 26G × 13 mm Microlance™ hypodermic needles and 75 × 1.15 mm heparinised capillary tubes. Blood samples were centrifuged immediately (10 min at 6000 RPM) to separate red blood cells from plasma. Plasma and red blood cells were pipetted into separate vials and stored on ice until they could be transferred to a − 20 °C freezer on the same day. Once fieldwork was complete, all samples were sent to the lab and stored at − 80 °C until analysis.

### Insect monitoring

Flying insects were trapped using flight intercept traps hung from trees from May to August in each year. Four traps were placed at each of two sites with high spotted flycatcher occupancy. Two of these traps were hung adjacent to a river and two were hung approximately 80 m from the same river. Insects were trapped in 1 L bottles containing 60 mL propylene glycol, 40 mL water and a drop of liquid detergent. Traps were emptied weekly and insects were decanted through a 1 mm sieve and stored in 100% ethanol until sorting and identification. Non-flying insects were discarded and the remaining insects were identified to order level and their body length was measured ± 1 mm. Due to time constraints, it was not possible to identify insects to family level, and so we considered the effect of three exclusively aquatic insect orders Ephemeroptera, Plecoptera and Trichoptera (EPT) versus all other insects. Biomass was later calculated using allometric equations based on order and body length (Hódar 1996; Ganihar 1997; Sabo et al. 2002).

### Fatty acid extraction and gas chromatography/mass spectrometry (GC/MS)

Fatty acids were extracted from blood plasma using established methodology (Andersson et al. 2015; Eikenaar et al. 2022). Briefly, lipid extraction of up to 5 μL plasma was performed for 1 h using 50 μL of chloroform:methanol (2:1 v/v) with 33.3 ng/μL of internal standard methyl cis-10-heptadecenoate (purity > 99%, Aldrich). Base methanolysis was then used to convert fatty acid moieties in the solvent to fatty acid methyl esters (FAMEs) by adding 100 μL of 0.5 M KOH in methanol and placing samples in the oven for 1 h at 40 °C. The reaction was halted by neutralising the base with 100 μL 0.5 HCl in methanol and FAMEs were separated by adding 300 μL heptane. The polar layer was removed and samples were washed twice with H_2_O, with the removal of the polar layer between each wash. Then, the heptane including the FAMEs was dried with anhydrous sodium sulphate before concentrating the heptane phase under N_2_. The FAME extracts were then quantified using Agilent 5975 MS coupled to an Agilent 6890 GC equipped with an HP-INNOWax column (30 m × 0.25 mm i.d., and 0.25 µm film thickness; Agilent). In total, 20 fatty acids were identified by comparing mass spectra with known retention times of synthetic standards.

### Data handling and statistical analyses

All statistical analyses were carried out in R version 4.3.1 (R Core Team 2020) using the Bayesian package *brms* (Bürkner 2021). In all cases, model fit was checked by comparing the posterior predicted distribution to the observed data, and chain convergence was assessed visually and using the R-hat value. Default priors were used for all models, and hypothesis testing was based on model estimates and the overlap of 90% credible intervals with 0. Four chains were run for 4000 iterations with a burn-in of 1000 and a thinning interval of 1. Effect sizes are reported as the median of the posterior distribution and 90% CRIs, unless otherwise stated.

#### Fatty acid analysis

Fatty acid composition was calculated for 84 nestling and 14 adult spotted flycatchers (6 females and 8 males). Chicks were sampled from 25 of 44 successfully hatched broods. Of the adult sample, eight were from pairs (4 broods) and six were individuals (6 broods). All but four of the adults from three different broods were parents of chicks that were sampled, but were sampled at different times than the chicks. We calculated relative proportions for the 20 fatty acids by dividing the area for each individual fatty acid peak by the sum of all fatty acid peaks (Table S1). Absolute fatty acid concentration was calculated by dividing the fatty acid peak area by the internal standard peak area, then multiplying by the internal standard concentration (33.3 ng/µL), and finally dividing by the sample plasma volume. Here, we present the absolute concentrations of highly unsaturated ω-3 fatty acids eicosapentaenoic acid (EPA), docosahexaenoic acid (DHA) and the ω-6:ω-3 ratio.

To test for correlations between ω-3 LC-PUFAs and ω-6:ω-3 with body condition, we used generalised linear (adults) and generalised linear mixed models (chicks) fitted with a Gamma distribution and log link function. Brood ID was included as a random effect in models for chicks to account for non-independence amongst individuals within the same nest. We created two models investigating the relationship with body condition: one for the response to ω-3 LC-PUFA concentrations (EPA and DHA) and one for the response to ω-6:ω-3.

To investigate the influence of river proximity on fatty acid concentrations, we constructed individual GLMMs for EPA, DHA and the ω-6:ω-3 ratio which included age (adult/chick), river proximity (m) and year as fixed effects and brood ID as a random effect. All models were fitted using a Gamma distribution and log link function.

#### Breeding success analysis

We tested the relationship between breeding success and aquatic insect availability using four breeding success metrics: clutch size, number of hatchlings (number of eggs known to have hatched), fledging success (a binary measure of whether at least one chick fledged from a sampled nest), and the number of chicks fledged (the number of chicks present 1–3 days prior to fledging). Clutch size is known to be correlated with first egg date (FED) (Stevens et al. 2007) and so FED was included as a fixed effect in the clutch size model. There was a relatively strong correlation between FED and both aquatic and terrestrial insect biomass (Pearson’s correlation − 0.50 and − 0.63, respectively), and so we compared three clutch size models that included FED (FED-only model), aquatic biomass + terrestrial biomass (insect-only model) or FED + aquatic biomass + terrestrial biomass (insect and FED model), and present the results for the most parsimonious model. We included year as a factor in all models to account for environmental differences between years. For all analyses, we excluded nests that were known to have been predated, i.e. the nest was found destroyed or empty before fledging was due.

**Fig. 1:**
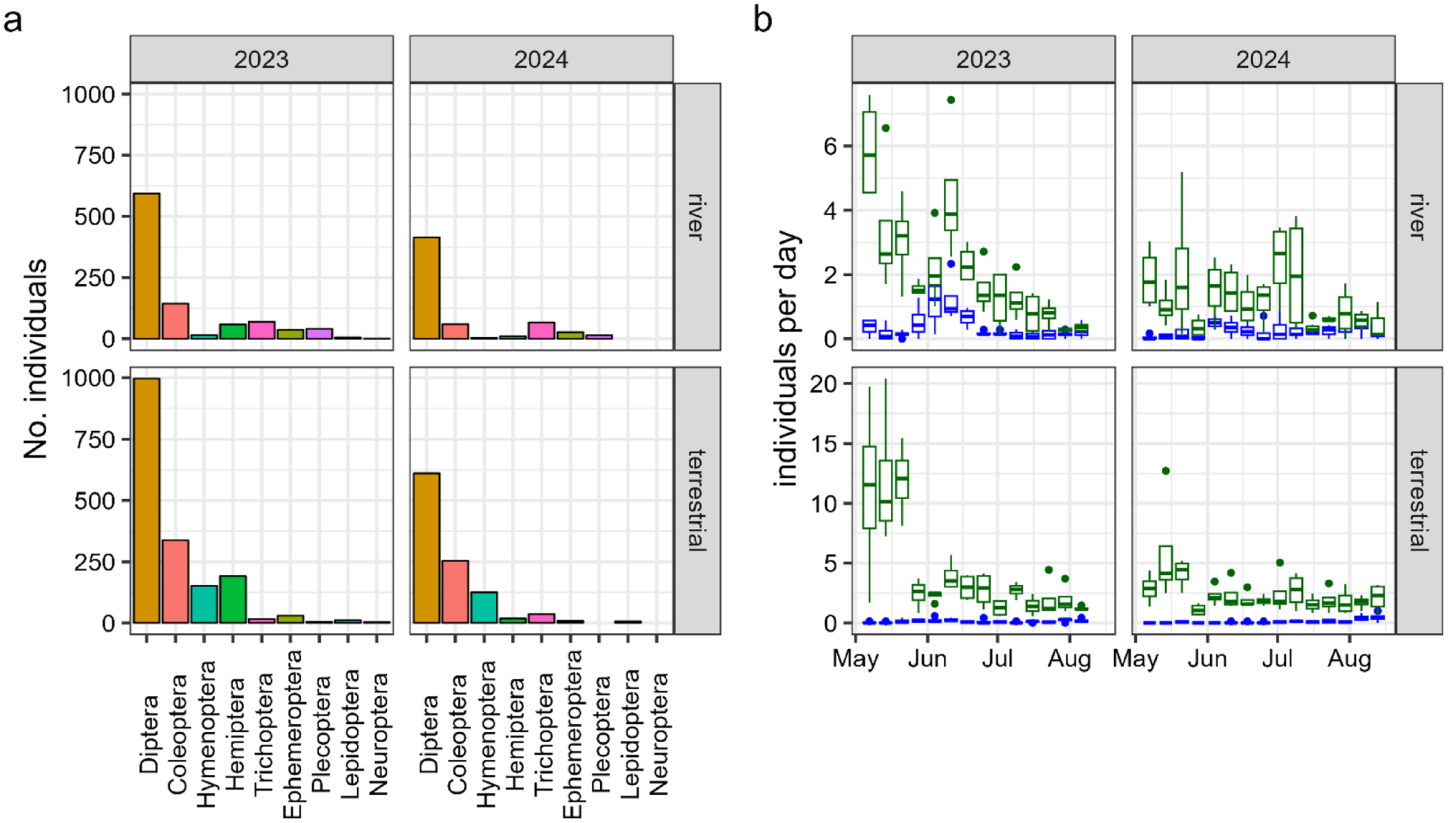
Trends in insects captured at traps located 80 m from a river (terrestrial panels) and immediately adjacent to a river (river panels) in Sedbergh during the 2023 and 2024 spotted flycatcher breeding seasons. Only insects > 2 mm in length are included. **a** Raw counts of each insect order by trap type, and **b** weekly trends in EPT taxa (blue) and all other insects combined (green) for river and terrestrial traps. Boxes display the median, first and third quartiles. Whiskers represent the largest and smallest values within 1.5 * interquartile range

Daily EPT and terrestrial insect availability was estimated using data from flight intercept traps. Insects < 2 mm were removed from the analysis as these were mostly unfavourable Diptera (e.g. Nematocera) which are unlikely to be eaten by spotted flycatchers (Davies 1977) and had a strong influence on the overall biomass due to large emergences following heavy rainfall at some sites. Weekly samples were standardised by dividing the biomass of insects by the length of time the trap was active. As both sites showed similar temporal trends, we pooled data into two groups—terrestrial (traps 80 m from a river) and river catches (Fig. 1). We then calculated a 7-day moving average to estimate daily availability of EPT and other insects for every day between the first and last insect sample. We used EPT data from river traps for the analyses as EPT taxa are most frequently found within 3 m of a river (Muehlbauer et al. 2014), evidenced by almost double the amount of EPT being caught in river traps compared to terrestrial traps (Fig. 1b, Table S2). Similarly, catches of other insects were 1.4 times higher in terrestrial traps, and so we assumed that variation in their availability would be most reliably reflected by data from terrestrial traps (Fig. 1b, Table S3).

The daily sum of insect biomass during the life stage associated with each breeding success metric was calculated (Table 1). The correlation between insect biomass and each breeding success metric was then modelled with the appropriate distribution (Table 1). We compared the performance of models using separated insect groups (EPT + other insects) with models using the summed total of EPT from river traps and other insects from terrestrial traps, to explore whether any observed effects were a result of total biomass or specific insect types. Interactions were only included where 90% credible intervals (CRIs) did not overlap 0.

**Table 1:** Model-fitting distributions, sample sizes and corresponding period for which insect biomass was calculated for GLMs testing the effect of insect biomass on clutch size, hatching success, fledging success and number of fledglings

Breeding metric (*n*)	Insect data period	Model-fitting distribution	Fixed effects tested
Clutch size (63)	7 days prior to FED	Gaussian	Year, first egg date, aquatic insect biomass, terrestrial insect biomass
Hatching success (42)	FED to 1 day prior to hatch date	Weighted binomial	Year, aquatic insect biomass, terrestrial insect biomass, aquatic insect biomass*distance from river
Fledging success (59)	Hatch date to 13 days after hatch date	Bernoulli	
Number of fledglings (30)	Hatch date to 13 days after hatch date	Weighted zero-inflated binomial	

Weighted binomial models were all weighted with clutch size

FED = first egg date

## Results

### Fatty acids

#### ω-3 LC-PUFA concentrations

**Fig. 2:**
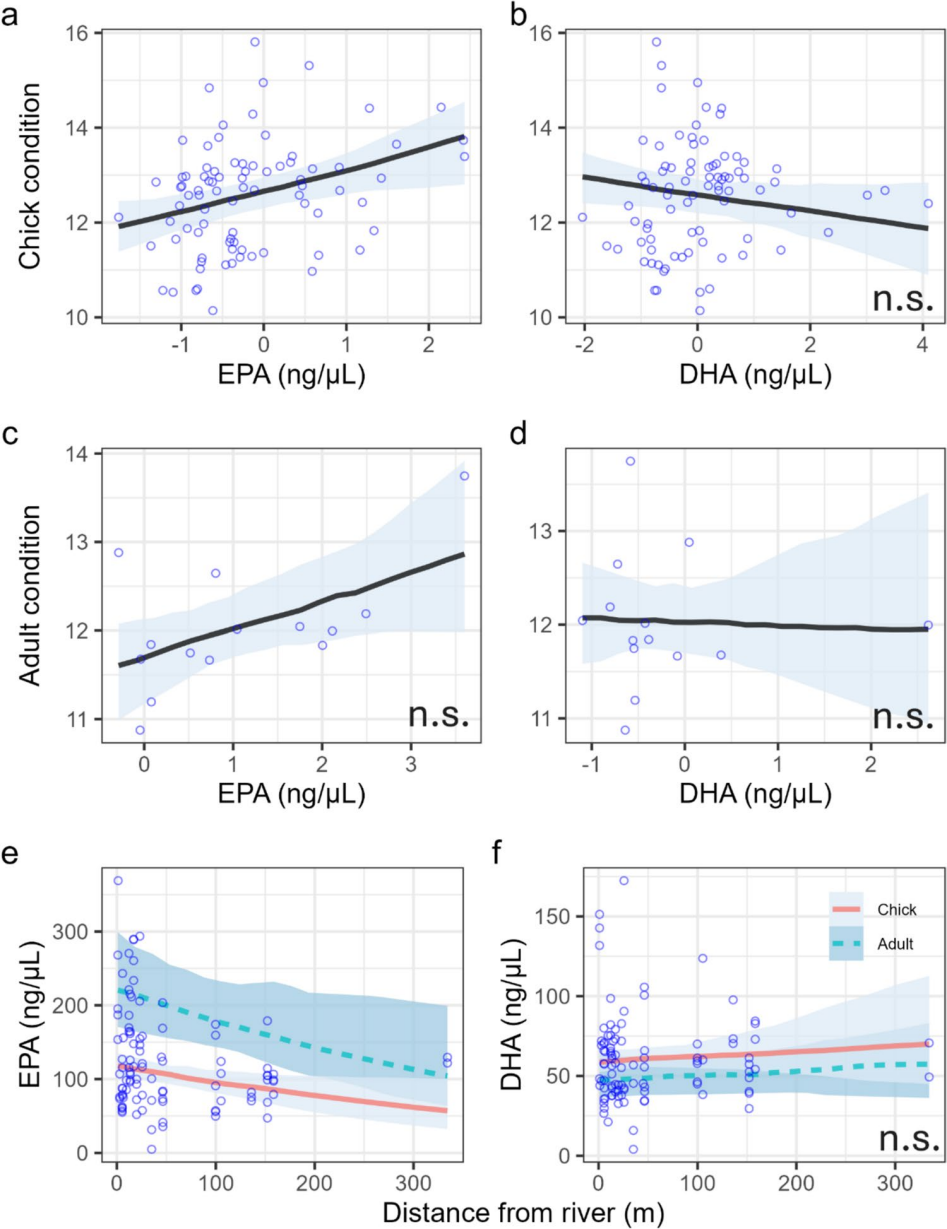
Highly unsaturated fatty acid (ω-3 LC-PUFA) concentration is correlated with the body condition of spotted flycatcher chicks (**a**,**b**), adults (**c**,**d**) and proximity to rivers (**e**,**f**). Body condition was calculated as a scaled mass index of tarsus length and mass (see “Methods” for details). Open circles show raw data; solid lines and shaded areas represent the median of the expected posterior predicted distribution and 90% credible intervals (CRIs). ‘n.s.’ denotes non-significance, i.e. 90% CRIs of the main effect (*x* axis) overlapping 0. In plots **e**, **f**, unbroken orange lines represent chicks and broken blue lines represent adults. The *x* axes in plots **a**–**d** show mean-centred values, so negative values indicate observations below the mean. EPA—eicosapentaenoic acid, DHA—docosahexaenoic acid

Chick condition was positively correlated with plasma EPA concentration (effect size: 0.04, 0.01–0.06) but not DHA concentrations (effect size: − 0.01, − 0.03 to 0.00; Fig. 2a–b, Table 2). Adults showed a similar trend but 90% CRIs overlapped 0 for both ω-3 LC-PUFAs (Fig. 2c–d, Table 2). EPA concentration was higher in chicks than adults (effect size: − 0.63, − 0.89 to − 0.37), ranging from 4.92 ng/μL to 289.59 ng/μL in chicks (mean: 112.37 ng/μL) and 104.88 ng/μL to 368.87 ng/μL (mean: 196.40 ng/μL) in adults (Fig. 2e, Table 2). The opposite was found for DHA, which ranged from 4.05 ng/μL to 172.40 ng/μL in chicks (mean: 61.10 ng/μL) and 29.84 ng/μL to 131.78 ng/μL (mean: 53.48 ng/μL) in adults, though there was considerable overlap between the two (Fig. 2f, Table 2). EPA concentration clearly decreased as distance from the river increased (− 0.14, − 0.26 to − 0.02) but no effect of river proximity was found for DHA (Fig. 2e–f, Table 2).

**Table 2:** Summary of results for Bayesian generalised linear and generalised linear mixed models testing the relationship between spotted flycatcher body condition and fatty acid concentration, and the relationship between fatty acid concentration and proximity of nests to rivers

Chick condition	Estimates	CRI (90%)	Marginal *R*^2^/conditional *R*^2^
**Intercept**	**2.54**	**2.52** to **2.56**	0.104/0.496
DHA (ng/μL)	− 0.01	− 0.03 to 0.00	
**EPA (ng/μL)**	**0.04**	**0.01** to **0.06**	
**Random effects**			
σ^2^	0.52		

Adult condition	Estimates	CRI (90%)	*R*^2^ Bayes
**Intercept**	**2.46**	**2.41** to **2.50**	0.352
DHA (ng/μL)	0	− 0.04 to 0.04	
EPA (ng/μL)	0.03	0.00 to 0.06	

EPA (ng/μL)	Estimates	CRI (90%)	Marginal *R*^2^/conditional *R*^2^
**Intercept**	**5.31**	**5.09** to **5.53**	0.262/0.622
**Distance from river**	− **0.14**	− **0.26** to − **0.02**	
**Age: chick**	− **0.63**	− **0.85** to − **0.42**	
**Random effects**			
σ^2^	2407.87		

DHA (ng/μL)	Estimates	CRI (90%)	Marginal *R*^2^/conditional *R*^2^
**Intercept**	**3.88**	**3.67** to **4.11**	0.039/0.258
Distance from river	0.04	− 0.05 to 0.14	
Age: chick	0.23	0.00 to 0.45	
**Random effects**			
σ^2^	186.35		

Chick condition	Estimates	CRI (90%)	Marginal *R*^2^/conditional *R*^2^
**Intercept**	**2.53**	**2.51** to **2.55**	0.080/0.458
**Ω6:Ω3**	− **0.03**	− **0.04** to − **0.01**	
**Random effects**			
σ^2^	0.49		

Adult condition	Estimates	CRI (90%)	*R*^2^ Bayes
**Intercept**	**2.49**	**2.45** to **2.52**	0.067
Ω6:Ω3	− 0.01	− 0.05 to 0.03	

Ω6:Ω3	Estimates	CRI (90%)	Marginal *R*^2^/conditional *R*^2^
**Intercept**	**0.3**	**0.20** to **0.41**	0.163/0.674
**Distance from river**	**0.09**	**0.02** to **0.16**	
Age: chick	− 0.07	− 0.17 to 0.02	
**Random effects**			
σ^2^	0.07		

All models including chick data were modelled with a brood ID random effect to account for repeat sampling. Bold denotes 90% CRIs not overlapping 0

#### ω-6:ω-3

**Fig. 3:**
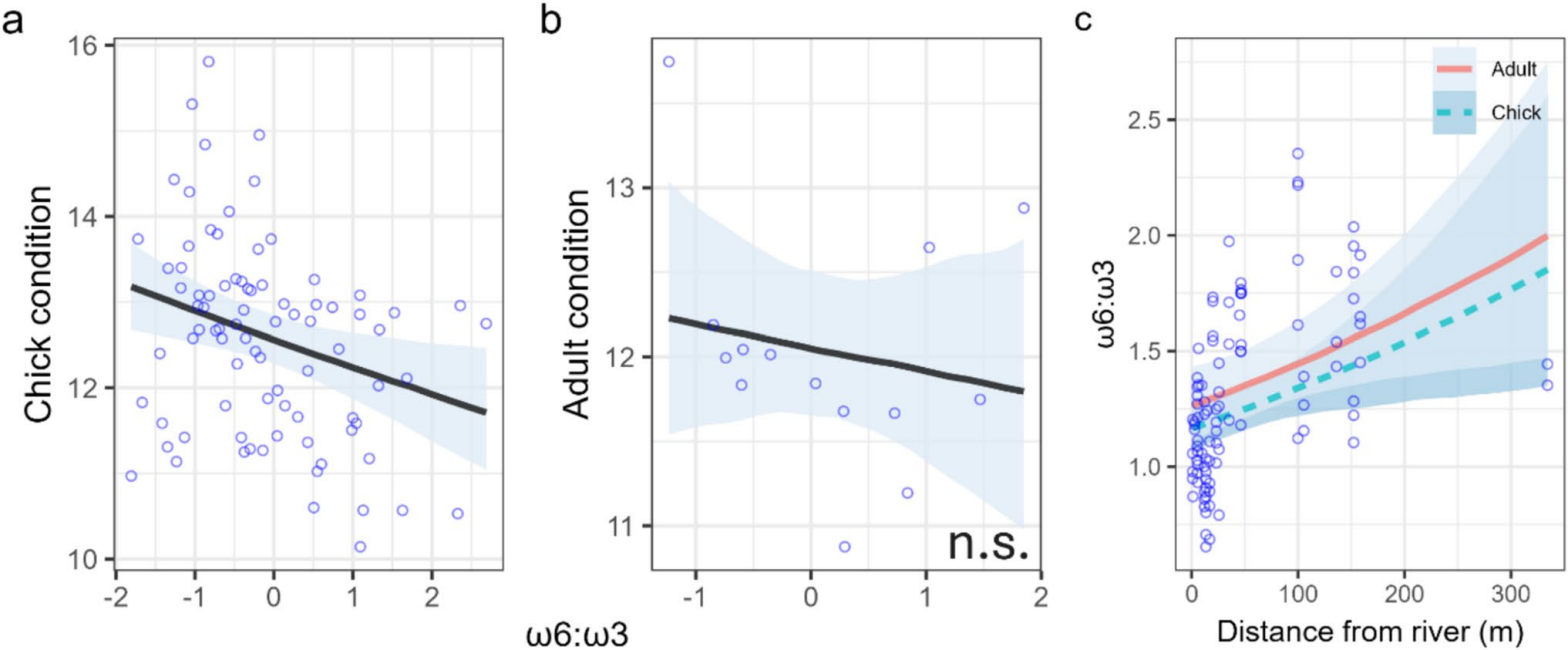
Correlation between omega-6:omega-3 (ω-6:ω-3) ratio and body condition (**a**,**b**) and river proximity (**c**) in spotted flycatcher chicks and adults. Body condition was calculated as a scaled mass index of tarsus length and mass (see “Methods” for details). Blue circles represent raw data. Lines and shaded areas represent the median of the expected posterior predicted distribution and 90% credible intervals (CRIs). ‘n.s.’ denotes non-significance, i.e. 90% CRIs of the main effect (*x* axis) overlapping 0. *X* axes in plots **a**–**b** are mean-centred values, so negative values indicate observations below the mean. In (**c**), the continuous orange line represents the predicted trend for adults and the dashed blue line represents chicks

The ratio of ω-6:ω-3 had a negative correlation with chick body condition (− 0.03, − 0.04 to − 0.01), but not with adult condition (Fig. 3a–b, Table 2). No distinct difference was observed between the ω-6:ω-3 ratio in chicks and adults, which ranged from 0.65 to 2.35 (mean: 1.30) in chicks and 0.87 to 2.04 (mean: 1.40) in adults (Fig. 3c, Table 2). River proximity had a strong correlation with ω-6:ω-3, which increased with distance from the river (0.09, 0.02 to 0.16; Fig. 3c, Table 2).

### Breeding success

**Fig. 4:**
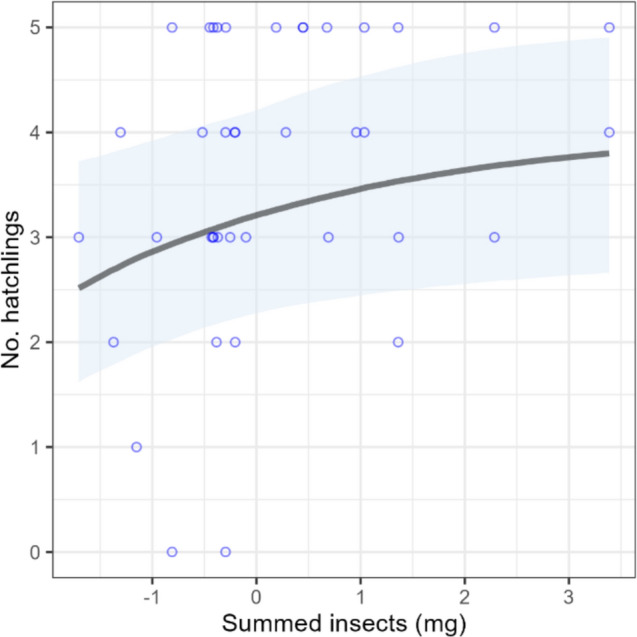
The predicted effect of insect biomass during the incubation period of spotted flycatchers on the rate of hatching success, after controlling for clutch size. Lines and shaded areas represent the median of the expected posterior prediction and 95% credible intervals (CRIs). Blue circles represent raw data. The *x* axis shows mean-centred values, so negative values indicate observations below the mean

**Fig. 5:**
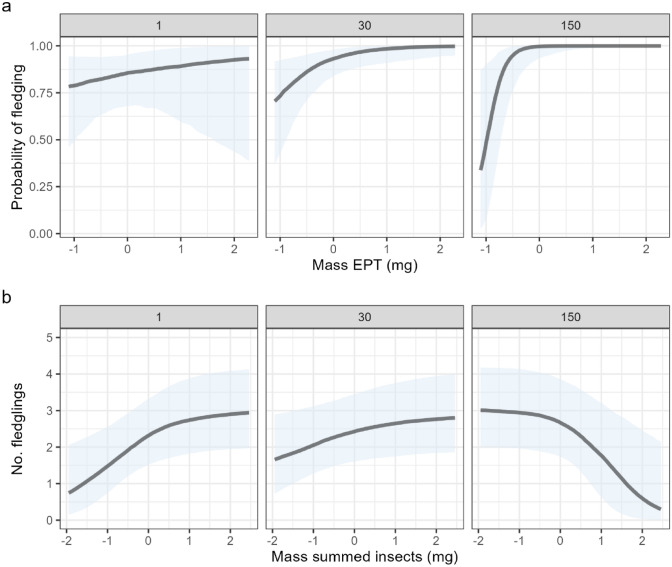
The predicted probability of spotted flycatcher nests fledging (**a**) and the number of fledglings (**b**) in relation to the mass of Ephemeroptera, Plecoptera and Trichoptera (EPT) and river distance during the nestling period. Lines and shaded areas represent the median of the expected posterior prediction and 95% CRIs. Titles above each plot represent the distance from the river (m) for which the prediction was made. All *x* axes show mean-centred values, so negative values indicate observations below the mean

In total, 128 nesting attempts were monitored between 2023 and 2024. Of those, 31 were predated and so were excluded from these analyses. Complete clutch size was known for 63 nests and was included in the clutch size analyses. First egg date was the most parsimonious predictor of clutch size. The FED-only model accounted for more variation in clutch size than the insect-only model (Bayes *R*^2^: 64.7 vs 0.26) and performed equally to the insect + FED model (Bayes *R*^2^: 64.8). Of 80 nests that successfully hatched, 42 had sufficient data (i.e. number hatched and completed clutch size) to be included in the hatching success analyses. We found no evidence that EPT biomass during the incubation period influenced hatching success, but the summed total of insects was positively correlated with hatching success (Fig. 4, Table 3). Of 71 nests that hatched successfully and were not subsequently predated, 59 had sufficient data to be included in the fledging success analysis. EPT biomass during the chick growth stage was positively correlated with fledging success, depending on how close the nest was to a river (Fig. 5, Table 3). Greater EPT biomass resulted in a greater probability of fledging at least 1 chick, and the strength of this effect was greater further away from rivers (Fig. 4a, Table 3). The relationship was similar, but had a smaller effect size, for an interaction between river distance and summed insects (Supp. Table 2). The sample size for the number of fledglings was smaller (*n* = 30) due to accessibility constraints and confidence in the number of fledglings within each nest during the final days pre-fledging. Nonetheless, there was a significant interactive effect of EPT biomass and river distance on the number of fledglings (Supp. Table 2), but the interaction between summed insect biomass and river distance had a greater effect size (Fig. 5, Table 3). The effect of summed insect biomass on the number of fledglings was greater when the nest was closer to a river (Fig. 4b, Table 3); however, the effect was positive for both fledging success and the number of fledglings up until approximately 50 m from a river (Figs. S2, S3).

**Table 3:** Summary of models testing the relationship between insect availability and four different breeding success variables

Predictors	Estimates	CRI (90%)	*R*^2^ Bayes
*Clutch size*			
**Intercept**	**5.81**	**5.54** to **6.07**	0.655
**egg1**	− **0.03**	− **0.04** to − **0.03**	
**Year: Year 2024**	− **0.31**	− **0.50** to − **0.13**	

Hatchlings	Log-odds	CRI (90%)	*R*^2^ Bayes
**Intercept**	**1.41**	**1.01** to **1.85**	0.296
Year: year 2024	0.53	− 0.22 to 1.31	
**Mass of summed insects**	**0.45**	**0.10** to **0.86**	

Fledging success	Log-odds	CRI (90%)	*R*^2^ Bayes
**Intercept**	**3.34**	**2.02** to **5.30**	0.172
Year: year 2024	− 0.97	− 2.32 to 0.29	
**Mass EPT during growth period**	**2.35**	**0.76** to **4.68**	
**Distance from river**	**2.15**	**0.34** to **5.32**	
Mass of other insects during growth period	− 0.72	− 1.54 to 0.00	
**Mass EPT during growth period: distance from river**	**2.69**	**0.33** to **6.26**	

Number of fledglings	Log-odds	CRI (90%)	*R*^2^ Bayes
**Intercept**	**1.62**	**0.93** to **2.39**	0.14
Year: year 2024	− 0.28	− 1.21 to 0.67	
Distance from river	0.52	− 0.13 to 1.47	
Mass summed of insects during growth period	0.19	− 0.34 to 0.72	
**Mass summed of insects during growth period: distance from river**	− **1.50**	− **2.52 to **− **0.58**	

Models were fit using Bayesian generalised linear mixed models in brms. Bold denotes 90% CRIs not overlapping 0

## Discussion

Our results show that access to dietary ω-3 LC-PUFAs during the breeding season was positively correlated with the condition and breeding success of a non-aquatic, generalist insectivorous bird. Variation in EPA concentration and ω-6:ω-3 in chicks and adults was explained by distance to a river, suggesting a substantial contribution of aquatic insects to these values, and this was supported by a higher fledging success at nests exposed to a greater biomass of aquatic insect orders. The apparent absence of an effect of DHA, either on body condition or relative to river proximity, was contrary to our expectations and is discussed below. These results support previous findings that show river density was positively correlated with spotted flycatcher occupancy (Eden 2025) and suggest that the results may at least be partly explained by improved breeding success resulting from access to better food quality and not just a potential consequence of variation in habitat structure adjacent to rivers.

Our confirmation that body condition in both chicks and adults increased with concentrations of EPA and lower ω-6:ω-3 was in line with expectations. Twining et al. (2016b) showed that, under laboratory conditions, tree swallow chicks fed low amounts of a high ω-3 LC-PUFA diet were heavier than chicks fed a large amount of a low ω-3 LC-PUFA diet, suggesting that quality is more important than quantity, at least during early development. However, various other factors contribute to chick development in the wild, e.g. predation risk and parental quality (Remeŝ and Martin 2002), which could mask or dilute the benefits of a high-quality diet. Our results show that EPA concentration in wild birds is positively related to body condition, a commonly used proxy for health and post-fledging survival (Naef-Daenzer et al. 2001; Ronget et al. 2018; Evans et al. 2020). Moreover, fledging success was greater in nests exposed to a higher biomass of EPT during the chick development period, suggesting that the positive effect of dietary ω-3 LC-PUFAs extends beyond the individual level.

It is likely that the limited sample size in the adult models (*n* = 14 of 10 broods) reduced the power to detect an effect, although the trend was the same as that for chicks. The influence of ω-3 LC-PUFAs on adult birds has been largely overlooked in the context of aquatic subsidies, but the potential importance of ω-3 fatty acids for migration has been demonstrated in other species (Weber 2009). For example, semipalmated sandpipers (*Calidris pusilla*) switch to feeding exclusively on DHA-rich prey prior to migration, which is thought to improve migration performance through increased metabolic efficiency and endurance in flight muscles (Weber 2009). In addition, improved body condition in adults with higher EPA concentrations may enhance survival (Blums et al. 2005) or breeding success (Kouba et al. 2021). These studies highlight the importance of a high-quality diet throughout the lifecycle, and future research should aim to explore the long-term impact of a high-quality diet on adult insectivorous birds.

The apparent lack of an effect of DHA on body condition in either adults or chicks, and the lack of a correlation with river proximity, was surprising given the importance of DHA in brain development and functioning. However, demand for DHA during the early developmental stages is high in altricial species, which undergo rapid brain growth between hatching and fledging (Speake and Wood 2005), and so is likely highly regulated. Whilst aquatic insects contain higher concentrations of DHA than terrestrial insects, Twining et al. (2018a) calculated that this amount was still insufficient to meet dietary demand, and so a large proportion of DHA is likely acquired through elongation of EPA, which requires fewer biomechanical reactions and is more energetically efficient than conversion of DHA from ALA. Although aquatic insects provide higher DHA potential through a higher EPA contribution, conversion is likely constrained to necessary levels and DHA is immediately transported to where it is required (i.e. the brain), which would result in less variation in the blood pool (sampled in this study).

Differences in ω-3 LC-PUFA concentrations between chicks and adults may suggest preferential feeding of high-quality food items to chicks. Spotted flycatchers have been observed selectively feeding chicks larger insects (Davies 1977), but it is unclear whether specific orders or families with differing ω-3 LC-PUFA potential are selected. Significantly lower EPA concentrations and marginally higher DHA concentrations in chicks (Fig. 1e–f) may have resulted from preferential feeding of DHA to chicks by adults. However, high DHA demand during development may lead to higher EPA to DHA conversion in chicks compared to adults, which would lead to higher DHA and lower EPA concentrations in the blood. Thus, a more detailed understanding of EPA to DHA conversion and of the insect species eaten by adults and chicks is required to explain these trends.

The ω-6:ω-3 ratio had a similar effect on chick condition to EPA, which is unsurprising given the biological mechanisms influenced by ω-6:ω-3. First, ω-6 competes with ω-3 for binding sites (Twining et al. 2016a), and so higher ω-6:ω-3 ratios can lead to lower deposition of EPA and DHA in muscles (Ibrahim et al. 2018). Moreover, ω-6 is pro-inflammatory, whilst ω-3 is anti-inflammatory, and so the ratio between the two can determine the levels of inflammation and oxidative stress in the body (Larsson et al. 2004; Isaksson 2015). Here, we demonstrated that the proximity to rivers, a proxy for aquatic insect availability, was negatively associated with ω-6:ω-3. Though we were unable to trace the origins of fatty acids in our study (e.g. through isotope labelling), the relationship between plasma EPA and ω-6:ω-3 with river proximity implies that aquatic insects likely drove the variation in fatty acid content.

Although we did not measure the ALA to EPA conversion efficiency of spotted flycatchers, previous work has suggested that other generalist species are less likely to be limited by dietary ω-3 LC-PUFAs as they have better conversion efficiency than species reliant on aquatic resources (Twining et al. 2021). However, our results suggest that, although not limited, generalist species may still benefit from higher quality food. Most aquatic insect deposition occurs within 0.5 m of the river, though some species range further than 500 m (Muehlbauer et al. 2014). The maximum distance of a flycatcher nest from a river in our study was 334 m, which exceeds the average distance that spotted flycatchers travel from their nests to feed (Davies 1977), and so the lower EPA concentrations measured in samples furthest from rivers are likely to be the result of a combination of ALA conversion and a small contribution of aquatic insects. Chicks nearer to rivers in our study benefited from better access to aquatic insects and their high EPA content. They were in better condition than chicks that had access to a lower quantity of aquatic insects, suggesting potential for ω-3 LC-PUFA-rich aquatic subsidies to benefit insectivorous species, regardless of dietary niche.

The results of our breeding success analyses further emphasise the importance of aquatic subsidies for generalist insectivores. Hatching success did not appear to be driven by EPT availability, but was positively associated with the overall availability of insects. This suggests that during incubation, a plentiful supply of any prey type may be beneficial, potentially by reducing the frequency of foraging bouts and thereby improving egg temperature regulation and reducing predation risk. Conversely, fledging success within 50 m of rivers responded positively to higher EPT availability (Figs. S2, S3), which clearly demonstrates the benefit of having access to high-quality food during chick development. This difference in response between breeding stages may reflect the higher nutritional demands of growing chicks, with EPT taxa providing essential ω-3 LC-PUFAs that are critical for development (Speake and Wood 2005; Dyall 2015).

The driver of the interactions between river proximity and insect biomass on productivity is unclear, but may be a result of the study design. The abundance of EPT in this study most accurately reflects the availability of EPT to nests closer to rivers, as these orders are most likely to be found within 3 m of a river (Muehlbauer et al. 2014). The availability of EPT to nests further away from rivers is confounded by various factors including species-specific behaviour, river productivity and habitat composition (Muehlbauer et al. 2014). Indeed, EPT catch rate at our river traps was on average double that of terrestrial traps (Fig. 1b, Table S2). Whilst EPT are a widely recognised indicator of high-quality aquatic prey, they represent only part of the aquatic insect community. Other emergent taxa, such as chironomids, can occur in high abundance and may contribute substantially to the diets of insectivores, particularly at sites further from rivers (Muehlbauer et al. 2014). As these groups were not measured in this study, our results likely underestimate the total influence of aquatic subsidies on breeding success.

Beyond rivers, other wetland habitats that support emergent aquatic insects, such as lakes and ponds, may provide similar benefits for insectivores. For example, one study found that insect biomass was 25-fold higher around well-managed ponds (Lewis-Phillips et al. 2020) and Berzins et al., (2021) found that tree swallow recruitment and chick condition were positively related to the abundance of surrounding ponds. Given the relative ease of introducing ponds into the landscape, these features may offer an effective way to disproportionately benefit insectivorous populations.

Humans have strong potential to disrupt overall ω-3 LC-PUFA export from aquatic habitats via land use and climate change. Increased frequency of droughts and both agriculture and pollution have the potential to alter the abundance and community structure of aquatic invertebrates (Ledger et al. 2013; Van Dijk et al. 2013; Stenroth et al. 2015; Manning and Sullivan 2021; Powell et al. 2024), which may differ in their ω-3 LC-PUFA composition (Parmar et al. 2022). In addition, the peak of aquatic insect availability has become earlier in the season as a result of climate change (Shipley et al. 2022), increasing the pressure for consumers to synchronise with high-quality food peaks. As our study took place in an area of relatively high water quality (Powell et al. 2023—Fig. S6), within a limited distance from a river, studies incorporating a wider range of habitats and availability of aquatic subsidies are required to fully understand the consequences of disrupting the availability of high ω-3 LC-PUFA diets for generalist species in the wild.

Our study examined the role of aquatic insects and their nutritional composition on a terrestrial, Palearctic insectivorous bird. Though the ability to synthesise ω-3 LC-PUFAs endogenously, and so the requirement for dietary ω-3 LC-PUFAs, may vary between species, direct consumption may still be energetically favourable. As the spotted flycatcher is not exclusively riparian, it is likely that it has sufficient ability to synthesise ω-3 LC-PUFAs from precursors, yet benefits were still apparent from direct consumption, demonstrating significant potential for aquatic insects to improve breeding success via improved food quality. As our study site was one of high river density, resulting in a large sample of nests within 50 m of a river, future work should aim to extend this work to a wider gradient of aquatic habitat proximity. Moreover, extending this understanding to other insectivorous species that are currently undergoing rapid declines across Europe and North America would be beneficial. Gaining a better understanding of the consequences of limiting these resources may help to quantify the potential of introducing (e.g. pond creation) or improving (habitat management) aquatic systems as a conservation measure for terrestrial species.

## Supplementary Information

Below is the link to the electronic supplementary material.

Supplementary file1 (DOCX 2808 KB)

## Data Availability

The datasets used during the current study are available from the corresponding author on reasonable request.

## Code availability

N/A.
