# Supplementary material for: Cross-system transfer of fatty acids from aquatic insects supports terrestrial insectivore condition and reproductive success
Source: Oecologia. 2025 Nov 12;207(12):191. doi: 10.1007/s00442-025-05827-9 (PMC12612012; doi:10.1007/s00442-025-05827-9)
Supplement: Supplementary file 1 — Supplementary file1 (DOCX 2808 KB) [file 442_2025_5827_MOESM1_ESM.docx]

**
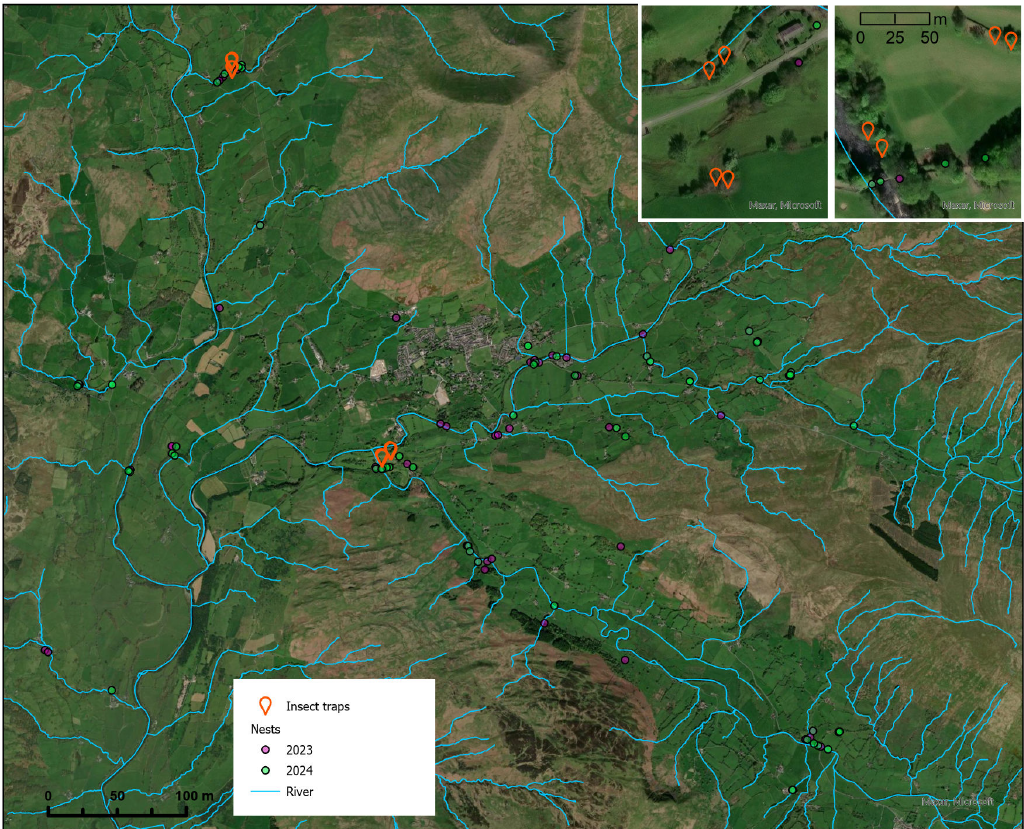
Figure S1.** Aerial view of study site in Sedbergh, Yorkshire Dales National Park, UK (54.323559, -2.528300). Monitored nests are shown as purple (2023) and green (2024) circles. Locations of insect traps are shown as red markers. The inset provides a close-up view of the insect traps, illustrating the open areas separating the river and terrestrial traps. Rivers are shown as blue lines. A large proportion of the area is roughly grazed upland, which is unsuitable for spotted flycatchers, Sedbergh was chosen as a study site due to its relatively high density of spotted flycatchers. Monitoring took place in the valleys of Sedbergh, which are predominantly utilised for pastoral farmland and rural settlements and are delineated by second- and third-order streams and rivers.

**Table S1**. Mean and SD of percentage of fatty acids in blood plasma of 14 spotted flycatcher adults and 84 7-day old chicks.

|  | **Mean (SD)** | | | |
| --- | --- | --- | --- | --- |
| **Fatty acid** | **Adults (%)** | | **Chicks (%)** | |
| 12:0 Lauric acid | 0.15 | (0.08) | 0.33 | (0.40) |
| 14:0 Myristic acid | 1.50 | (0.28) | 2.22 | (1.55) |
| 15:0 Pentadecanoic acid | 0.22 | (0.06) | 0.19 | (0.08) |
| 16:0 Palmitic acid | 18.66 | (1.85) | 20.47 | (3.58) |
| 16:1(n-9) Hypogeic acid | 0.36 | (0.09) | 0.41 | (0.17) |
| 16:1(n-7) Palmitoleic acid | 11.70 | (2.57) | 12.32 | (3.48) |
| 17:0 Heptadecanoic acid | 0.60 | (0.13) | 0.62 | (0.17) |
| 18:0 Stearic acid | 12.64 | (1.10) | 13.68 | (1.32) |
| 18:1(n-9) | 16.18 | (1.42) | 18.24 | (2.85) |
| 18:1(n-7) Vaccenic acid | 1.43 | (0.62) | 2.32 | (0.84) |
| 18:2(n-6) Linoleic acid | 11.95 | (2.19) | 9.26 | (1.73) |
| 18:3(n-3) Alpha-linolenic acid | 5.03 | (1.82) | 4.54 | (2.06) |
| 20:0 Arachidic acid | 0.14 | (0.06) | 0.18 | (0.21) |
| 20:1(n-9) Gondoic acid | 0.04 | (0.02) | 0.09 | (0.05) |
| 20:3(n-6) Dihomo-gamma-linolenic acid | 0.43 | (0.13) | 0.51 | (0.20) |
| 20:4(n-6) Arachidonic acid | 8.19 | (1.43) | 6.01 | (1.83) |
| 20:5(n-3) Eicosapentaenoic acid | 7.19 | (2.45) | 4.40 | (1.75) |
| 22:4(n-6) Adrenic acid | 0.23 | (0.44) | 0.13 | (0.05) |
| 22:5(n-3) Docosapentaenoic acid | 1.37 | (0.35) | 1.64 | (0.52) |
| 22:6(n-3) Docosahexaenoic acid | 1.99 | (0.82) | 2.45 | (0.92) |

**Table S2.** Output of linear random effects models comparing the different log1p transformed catch rates of EPT between terrestrial and river flight intercept traps monitored between May and August 2023 and 2024. Catch week and trap ID were included as random effects to account for baseline local and temporal variation in catch rates. Values have been back-transformed to original scale.

|  | **EPT catch rate** | | | |
| --- | --- | --- | --- | --- |
|  | **Estimates** | **CI** |  | ***p*** |
| Intercept | 0.24 | 0.18–0.30 |  | <0.001 |
| Type [terrestrial] | -0.14 | -0.20–-0.09 |  | <0.001 |
|  |  |  |  |  |
| **Random effects** |  |  |  |  |
| σ2 | 0.03 |  |  |  |
| τ00 [week] | 0.01 |  |  |  |
| τ00 [trap ID] | 0.00 |  |  |  |

**Table S3.** Output of linear random effects models comparing the different log1p transformed catch rates of ‘other insects’ (all flying insects excluding EPT) between terrestrial and river flight intercept traps between May and August 2023 and 2024. Catch week and trap ID were included as random effects to account for baseline local and temporal variation in catch rates. Values have been back-transformed to original scale.

|  |  | **‘Other insects’ catch rate** | | |
| --- | --- | --- | --- | --- |
|  | **Estimates** | **CI** |  | ***p*** |
| Intercept | 0.83 | 0.66-1.00 |  | <0.001 |
| Type [terrestrial] | 0.43 | 0.32-0.55 |  | <0.001 |
|  |  |  |  |  |
| **Random effects** |  |  |  |  |
| σ2 | 0.13 |  |  |  |
| τ00 [week] | 0.16 |  |  |  |
| τ00 [trap ID] | 0.01 |  |  |  |

**Table S4**. Summary of discarded models testing the relationship between insect availability and four different breeding success variables. Models were fit using Bayesian generalised linear mixed models in brms. Bold denotes 90% CRIs not overlapping 0.

| **Hatchlings** | *Log-Odds* | *CRI (90%)* | R^2^ Bayes |
| --- | --- | --- | --- |
| **Intercept** | **1.36** | **0.94 – 1.80** | 0.327 |
| year: year2024 | 0.65 | -0.14 – 1.48 |  |
| Mass EPT during laying period | 0.32 | -0.08 – 0.73 |  |
| Mass other insects during laying period | 0.21 | -0.24 – 0.69 |  |
|  |  |  |  |
| **Fledging success** | *Log-Odds* | *CRI (90%)* | R^2^ Bayes |
| **Intercept** | **2.53** | **1.59 – 3.76** | 0.102 |
| year: year2024 | -0.93 | -2.25 – 0.34 |  |
| distance from river | 0.72 | -0.17 – 2.02 |  |
| mass summed insects during growth period | 0.15 | -0.49 – 0.89 |  |
| **mass summed insects during growth period:distance from river** | **1.08** | **0.01 – 2.58** |  |
|  |  |  |  |
| **Number of fledglings** | *Log-Odds* | *CRI (90%)* | R^2^ Bayes |
| **Intercept** | **1.39** | **0.68 – 2.15** | 0.13 |
| year: year2024 | -0.13 | -1.13 – 0.89 |  |
| mass of EPT during growth period | 0.24 | -0.39 – 0.94 |  |
| distance from river | -0.01 | -0.74 – 0.91 |  |
| mass other insects during growth period | 0.13 | -0.43 – 0.70 |  |
| **mass EPT during growth period :distance from river** | **-1.27** | **-2.40 – -0.29** |  |
|  |  |  |  |

**Figure S2.** Predicted probability of fledging at least 1 chick from 0 to 300m from a river. Lines denote median of the posterior distribution and shaded areas represent 95% CRIs. Plot titles represent proximity to rivers (m). X axis has been mean-centred.


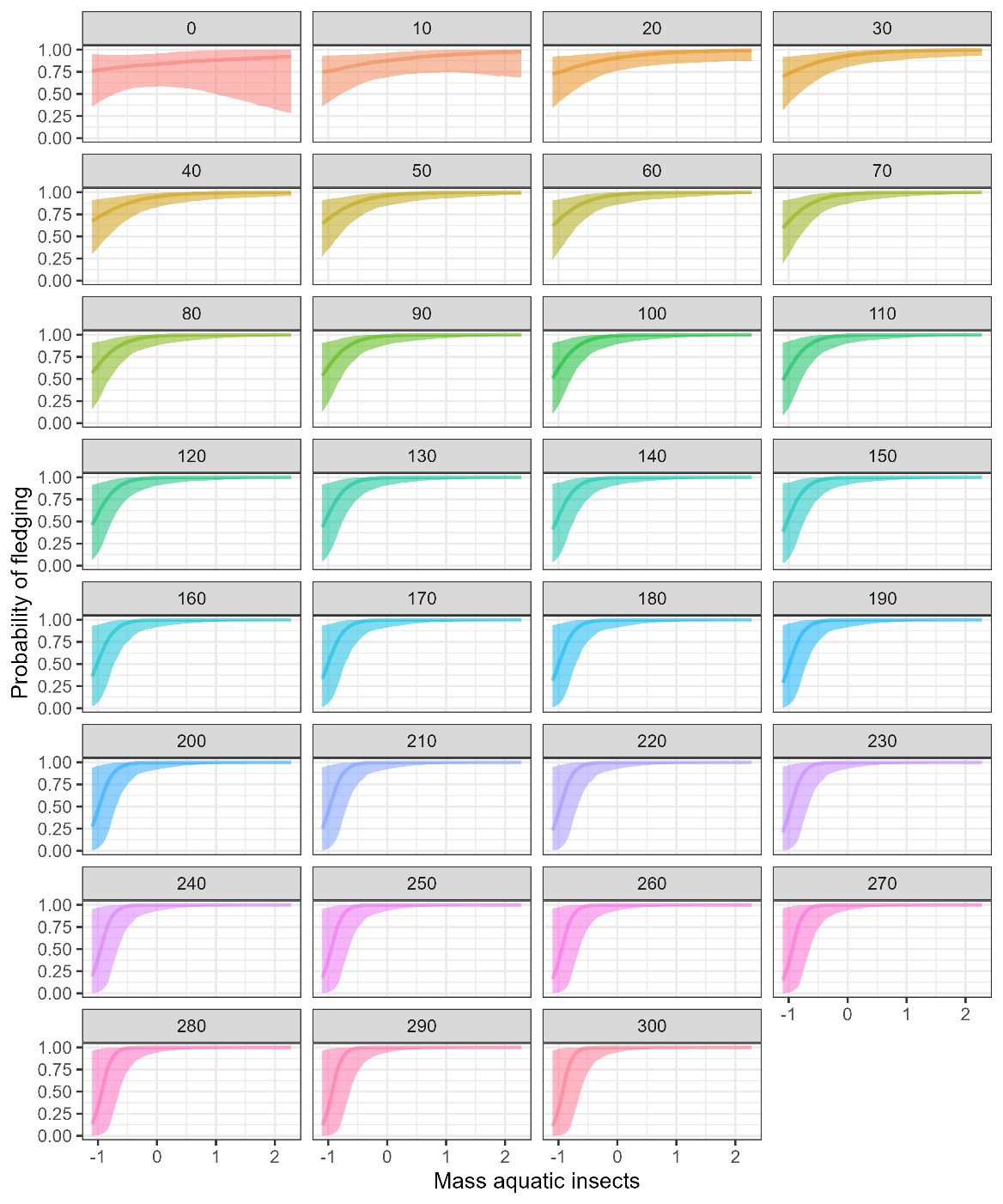

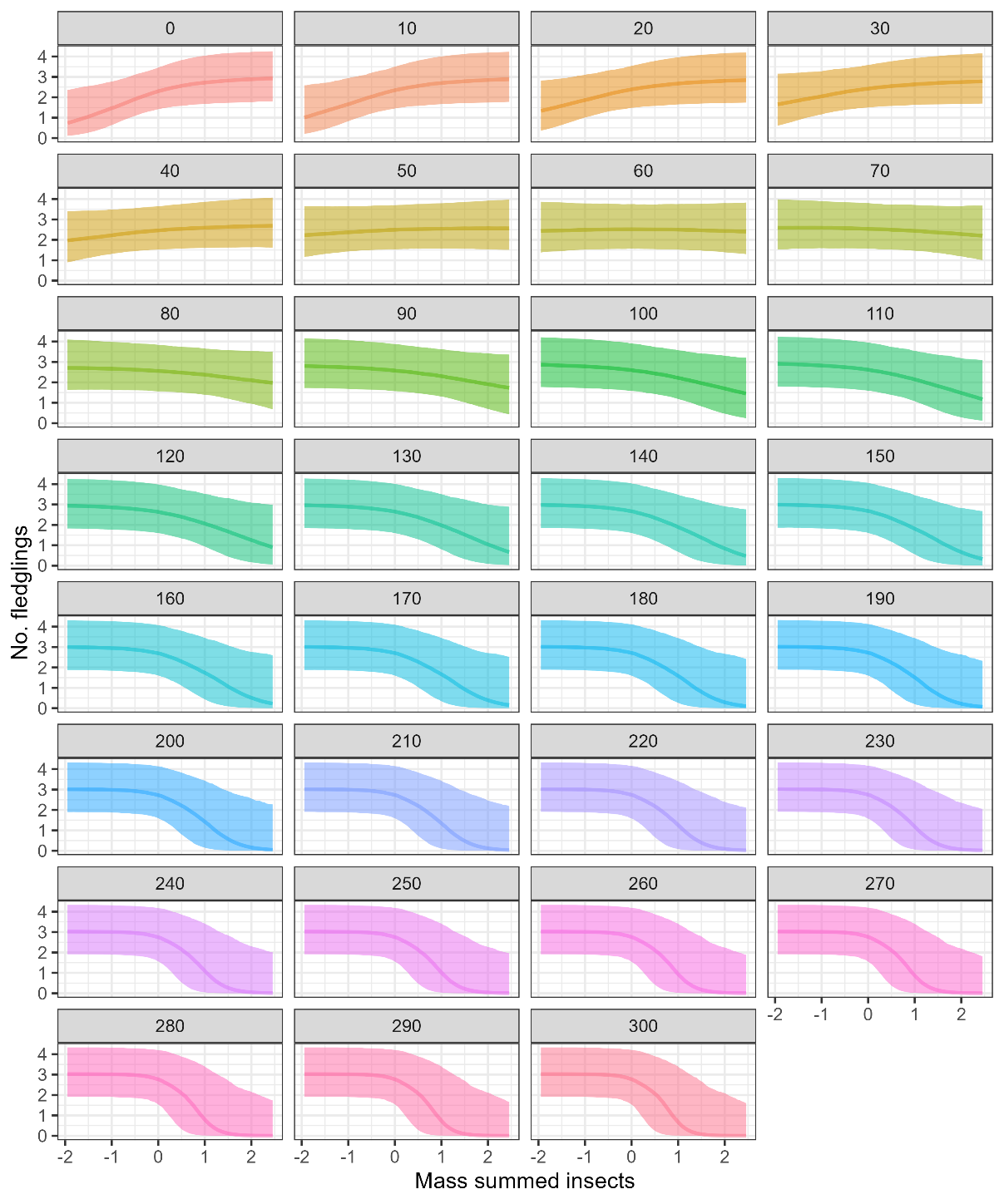


**Figure S3.** Predicted number of fledglings from 0 to 300m from a river. Lines denote median of the posterior distribution and shaded areas represent 95% CRIs. Plot titles represent proximity to rivers (m). X axis has been mean-centred.
